# Chemical composition and antimicrobial activity of Gannan navel orange (*Citrus sinensis* Osbeck cv. Newhall) peel essential oils

**DOI:** 10.1002/fsn3.688

**Published:** 2018-06-14

**Authors:** Qingyun Guo, Ke Liu, Weihui Deng, Balian Zhong, Wenxia Yang, Jiong Chun

**Affiliations:** ^1^ National Navel Orange Engineering Research Center College of Life and Environmental Sciences Gannan Normal University Ganzhou City Jiangxi Province China

**Keywords:** antimicrobial activity, chemical composition, essential oil, navel orange

## Abstract

The present investigation reported the chemical composition of cold pressed Gannan navel orange peel essential oil (EO) and its molecular distillation fraction (light phase EO), and examined their antimicrobial activity against spoiling and pathogenic microorganisms. Gas chromatography‐mass spectrometry analysis identified 27 and 20 different chemical constituents in cold pressed EO and light phase EO, respectively. Limonene was the major constituent, accounting for 85.32% of cold pressed EO and 60.44% of light phase EO. Both EOs and some of their constituents showed good antimicrobial activity. Compared to cold pressed EO, light phase EO exhibited the better antimicrobial activity under weak acidic and neutral conditions. The light phase EO presented a higher antimicrobial activity after thermo‐treatment at 60–100°C for 20 min than cold pressed EO. These results demonstrated that light phase EO had a potential to be used as a novel antimicrobial agent for food preservation and food processing.

## 1 INTRODUCTION

Citrus is one of the most important fruit tree crops in the world, and Brazil, China, and the United States are the world’s leading producers of citrus (USDA, 2017). The genus *Citrus* of the family Rutaceae includes more than 160 cultivated species distributed throughout the subtropical and temperate regions of China (Deng, 2008; Gmitter & Hu, 1990). Newhall navel orange is broadly cultivated in Gannan region of Jiangxi province in China. Gannan navel orange industry covers an area of 0.29 million acres with a total annual output of 1.2 million tons. Orange peel contains valuable essential oil (EO) which can be obtained using proper physical or chemical methods (Fisher & Phillips, 2008; Tongnuanchan & Benjakul, 2014). *Citrus* EO has a wide spectrum of antimicrobial activity against different groups of pathogenic organisms; therefore, it has been widely used in fields from food chemistry to pharmacology and pharmaceutics (Bakkali, Averbeck, Averbeck, & Idaomar, 2008; Sharma, Mahato, Cho, & Lee, 2017).

The composition of *Citrus* EO varies markedly according to variety, seasonality, geographic origin, ripeness of the fruit, extraction method, and also an interaction of various factors (Fisher & Phillips, 2008; Sharma et al., 2017). Usually, it is made up of a complex mixture of volatile (85%–99%) components (Moufida & Marzouk, 2003), including monoterpenes, sesquiterpenes, and their oxygenated derivatives (Raut & Karuppayil, 2014; Verzera, Trozzi, Gazea, Cicciarello, & Cotroneo, 2003). Limonene is the major chemical component of *Citrus* EO, ranging from 32 to 98% of the total oil (Moufida & Marzouk, 2003). The antimicrobial activity of EO is directly correlated to the presence of its bioactive volatile constituents, although it may be varied with environmental conditions (Bakkali et al., 2008; Perczak et al., 2016). The well‐known and characterized constituents of *Citrus* EO include limonene, linalool, and citral, which have been proved to exert potent, broad‐spectrum antimicrobial capacity (Bezic, Skocibusic, & Dunkic, 2005). As *Citrus* EOs are generally recognized as safe (GRAS), they have been screened for antimicrobial properties against common food‐borne pathogens (Bakkali et al., 2008; Burt, 2004). In addition, they have been used as natural food preservatives and accepted by consumers all over the world (Sharma et al., 2017).

In commercial practice, large‐scale orange EO is mainly prepared by cold pressing method (Leão, Sampaio, Pagani, & Silva, 2014). Cold pressed orange EO was extracted by mechanical rupturing of the oil sacs in the flavedo, expressing the oil as an aqueous emulsion from which it is separated by centrifuging. It consists of terpenes, alcohols, aldehydes, ketones, esters, and acids along with some nonvolatile waxy materials, carotenoids, flavonoids, etc. However, pigment, wax, and pesticide residue in EO are disadvantageous to be used as antimicrobial agents in food. Molecular distillation is an efficient method to separate some undesired molecules without harming the natural (desirable) properties of EO (Borgarello, Mezza, Pramparo, & Gayol, 2015). Separation can be achieved based on the difference of the mean free path of different molecules. This method is characterized by a high vacuum operation that causes a decrease in the boiling point of substances (Rossi, Pramparo Mdel, Gaich, Grosso, & Nepote, 2011). Therefore, molecular distillation is very useful to separate thermal sensitive EOs and extensively used in flavor and fragrance industry.

In this study, light phase EO was separated from cold pressed Gannan navel orange peel EO by molecular distillation. Then, we investigated the chemical composition of light phase EO and cold pressed EO and examined their effectiveness in vitro on four selected spoiling and pathogenic microorganisms.

## 2 MATERIALS AND METHODS

### 2.1 Extraction of cold pressed EO and light phase EO

Cold pressed EO was obtained by physical extraction from the peel of Gannan navel orange (*Citrus sinensis* Osbeck cv. Newhall) (Lv, Chun, Jiang, Li, & Guo, 2014). The recovery of orange oil by FOMESA extractor (Model 391, Food Machinery Espanola, S.A., Valencia, Spain) was carried out during simultaneous extraction of juice and oil. The oil cells were ruptured by pressure, and the oil was washed away with water. The oil emulsion passed through a 20‐mesh shaker screen to remove most of the insolubles. Then a three‐stage centrifuge system was used to separate orange oil efficiently. The first stage removed some water and insoluble solids by three‐phase disk stack centrifugal separator to produce an emulsion with around 40% oil content. This emulsion was fed to the second stage centrifuge, where the oil content was concentrated to around 88%. The third stage removed the remaining water and cloudy particles to achieve complete separation of the oil phase.

Light phase EO was obtained by molecular distillation from cold pressed EO using a wiped‐film molecular distillation apparatus (Pope Two Inch Laboratory Scale Wiped‐Film Molecular Still & Evaporator; Pope Scientific Inc., Saukville, WI, USA). The evaporation temperature and operation pressure were 50°C and 10.0 Torr, respectively. Orange oil was fed at room temperature, and the feeding rate was 3.0 ml/min. The rotational speed of the roller wiper was 350 rpm, and the condenser temperature was 0°C.

### 2.2 Chemicals

α‐Pinene (>98%), limonene (>98%), citral (>99%), decanal (>97%), and linalool (>98%) were purchased from Aladdin Chemical Reagent Co., Ltd., Shanghai, China. Nonanal (>96%) and terpineol (>96%) were purchased from Sigma‐Aldrich Chemical Reagent Co., Ltd., USA.

### 2.3 GC‐MS analysis

Gannan navel orange EO components were identified and quantified using an Agilent 7890B gas chromatograph coupled with an Agilent mass spectrometer detector. The GC‐MS system was equipped with a DB‐5 MS capillary column (30.00 m × 0.25 mm × 0.25 μm). Mass spectra were obtained by electron ionization (EI) at 70 eV with a spectra range of 50 to 500 m/z. The injector and detector temperatures were operated at 150°C and 250°C, respectively. The oven temperature was maintained at 80°C for 4 min and subsequently raised to 250°C (5°C/min) for 10 min. Helium was used as a carrier gas at a flow rate of 1.0 ml/min, at a split ratio of 100:1. Most of the components were identified by comparing their mass spectra with those of the computer mass libraries of The National Institute of Standards and Technology (2010).

### 2.4 Antimicrobial activity assays

#### 2.4.1 Microbial strains and growth conditions

The following microorganisms were purchased from China General Microbiological Culture Collection Center (CGMCC): *Escherichia coli* (ATCC25922), *Staphylococcus aureus* (ATCC25923), *Bacillus subtilis* (ATCC6633), *Saccharomyces cerevisiae* (ATCC204412). The first three microbial strains were maintained in Luria‐Bertani medium at 37°C, and the *S. cerevisiae* was maintained in Yeast Extract Peptone Dextrose (YEPD) medium at 25°C. Subsequently, one colony from each culture was inoculated in liquid medium for 18–24 hr with shaking (200 rpm) to obtain freshly cultured microbial suspensions (>10^8^ CFU/ml) for test.

#### 2.4.2 Determination of minimum inhibitory concentration (MIC)

MIC values of Gannan navel orange cold pressed EO, light phase EO, and seven individual constituents against microorganisms were determined by the tube dilution method (Burt, 2004; Rota, Carraminana, Burillo, & Herrera, 2004). Serial dilutions of EOs and individual constituents were prepared with liquid nutrient media containing 5 ml of LB or YEPD medium. Each tube was inoculated with 0.5 ml of a standardized suspension of microbial test species containing 1 × 10^6^ CFU/ml, then incubated at 37°C for 24 hr, except for *S. cerevisiae*, which was incubated at 25°C for 48 hr. The MIC was defined as the lowest concentration of EOs and individual constituents at which microorganisms failed to grow, so no visible changes were detected in the broth medium. All determinations were performed in triplicates.

#### 2.4.3 The effect of the environmental pH and temperature on antimicrobial activity of EOs

The effect of environmental acidity and alkalinity on the antimicrobial activity of EO was investigated according to the following method. The agar mediums were adjusted to pH value of 5.0, 6.0, 7.0, and 8.0 with 2% NaOH solution and 50% citric acid solution, and the mixtures were autoclaved at 115°C for 30 min. Cooled autoclaved agar was distributed evenly in Petri dishes aseptically. The antimicrobial activity of both EOs was determined using the agar disk diffusion method (Xin, Liu, Zhang, & Gao, 2016). Paper disks (6 mm) were impregnated with 5 μl of cold pressed EO and light phase EO and then placed on the inoculated Petri dishes containing tested microorganisms (1 × 10^6^ CFU/ml). The plates were incubated at 37°C for 24 hr, except for *S. cerevisiae*, which was incubated at 25°C for 48 hr. All determinations were performed in triplicates. Antimicrobial activity was evaluated by measuring the diameter of the inhibition zones to the nearest millimeter (mm). Sterile water alone was used as the control.

The antimicrobial activity of the thermo‐treated EOs was determined by the following method. Cold pressed EO and light phase EO were treated with heat at 60°C, 80°C, 100°C, and 121°C for 20 min, respectively. Then, paper disks (6 mm) were impregnated with 5 μl of heat‐treated EOs and the antimicrobial activity was determined according to the method previously described. The untreated EO stored at 25°C was used as the positive control. Sterile water alone was used as the negative control.

### 2.5 Statistical analysis

All data were expressed as the mean ± SD by measuring three independent replicates. Analysis of variance obtained was subjected to one‐way ANOVA. Means values were separated by Duncan’s multiple range tests when ANOVA was significant statistically (*p *< 0.05) (SPSS 18.0; Chicago, IL, USA).

## 3 RESULTS AND DISCUSSION

### 3.1 Chemical composition of essential oils

The chemical components of Gannan navel orange cold pressed EO and light phase EO were analyzed by GC‐MS (Table 1). As shown in Table 1 different chemical constituents, accounting for 98.73%, were identified in cold pressed EO. Similarly, 20 different chemical constituents, accounting for 93.17%, were identified in light phase EO which was obtained by molecular distillation from cold pressed EO. Limonene was the major constituent, accounting for 85.32% of cold pressed EO and 60.44% of light phase EO, consistent with previous studies that limonene was the main constituent of *Citrus* EO (Bakkali et al., 2008; Svoboda & Greenaway, 2003). Other compounds such as β‐myrcene (7.60%), α‐pinene (3.85%), carvone (3.30%), limonene 1,2‐epoxide (2.66%), linalool (2.26%), *cis‐p*‐mentha‐2,8‐dien‐1‐ol (2.31%), sabinene (2.23%), (*E*)‐carveol (2.00%), *tranns‐p*‐mentha‐2,8‐dien‐1‐ol (1.15%), and (*Z*)‐carveol (1.10%) contributed significantly to light phase EO final composition. For cold pressed EO, besides limonene, only β‐myrcene (5.11%), α‐pinene (1.95%), sabinene (1.12%), and linalool (1.29%) were present in amounts higher than 1%. Light phase EO has a higher content of oxygenated substances (17.28%) than cold pressed EO (4.25%).

**Table 1:** Chemical composition of Gannan navel orange cold pressed and light phase essential oils by GC‐MS

No.	RI	Compounds	Composition (%)	
			Cold pressed EO	Light phase EO
1	934	α‐Pinene	1.95	3.85
2	975	Sabinene	1.12	2.23
3	990	β‐Myrcene	5.11	7.60
4	1005	Octanal	0.57	—
5	1013	3‐Carene	0.53	0.73
6	1049	Limonene	85.32	60.44
7	1073	1‐Octanol	0.09	0.16
8	1104	Linalool	1.29	2.26
9	1108	Nonanal	0.13	0.14
10	1127	*trans‐p*‐Mentha‐2,8‐dien‐1‐ol	0.02	1.15
11	1137	Limonene 1,2‐epoxide	0.02	2.66
12	1144	*cis‐p*‐Mentha‐2,8‐dien‐1‐ol	—	2.31
13	1154	Citronellal	0.15	—
14	1200	α‐Terpineol	0.29	0.90
15	1209	Decanal	0.69	0.46
16	1223	(*E*)‐Carveol	0.04	2.00
17	1229	(*Z*)‐Carveol	0.05	1.10
18	1242	β‐Citral	0.16	0.13
18	1249	Carvone	0.04	3.30
19	1271	α‐Citral	0.24	0.18
20	1281	Perillaldehyde	0.07	0.31
21	1296	*p*‐Mentha‐1,8‐dien‐9‐ol	0.04	0.22
22	1379	α‐Copaene	0.12	—
23	1390	β‐Cubebene	0.13	—
24	1412	Dodecanal	0.15	—
25	1495	Valencene	0.17	0.36
26	1505	α‐Farnesene	0.03	0.68
27	1697	β‐Sinensal	0.13	—
28	1754	α‐Sinensal	0.08	—
Total			98.73	93.17

*Notes*

“—” indicates not be detected.

RI, retention indices determined on DB‐5 column, using the homologous series of *n*‐alkanes (C8–C20).

### 3.2 Antimicrobial activity

Cold pressed EO and light phase EO showed a wide spectrum of antimicrobial activity in vitro (Table 2). Regarding the Gram‐negative microorganisms (*E. coli*) studied, light phase EO (MIC = 0.78 μl/ml) showed better antimicrobial activity than that of cold pressed EO (MIC = 1.56 μl/ml). On the other hand, regarding the Gram‐positive bacteria (*B. subtilis* and *S. aureus*) tested, the bacteriostatic activity was weaker (MIC = 1.56–3.13 μl/ml), which may be due to the differing structures of their respective cell walls (Seow, Yeo, Chung, & Yuk, 2014). Regarding the eukaryotic microorganism (*S. cerevisiae*) tested, light phase EO showed higher antimicrobial activity (MIC = 0.39 μl/ml) than that of cold pressed EO (MIC = 1.56 μl/ml). Light phase EO showed more sensitivity to tested microorganisms under treatment conditions, and the low MICs (0.039–0.313% v/v) were suitable to food application. These results demonstrated that molecular distillation technology can provide EO fraction with better antimicrobial activity.

**Table 2:** Minimal inhibitory concentration (MIC) of Gannan navel orange essential oils (EOs) and their individual constituents

EOs and constituents	MIC values (μl/ml)			
	*Saccharomyces cerevisiae*	*Escherichia coli*	*Bacillus subtilis*	*Staphylococcus aureus*
Light Phase EO	0.39 ± 0.01^a^	0.78 ± 0.04^a^	1.56 ± 0.12^c^	3.13 ± 0.17^c^
Cold Pressed EO	1.56 ± 0.08^c^	1.56 ± 0.11^b^	1.56 ± 0.07^c^	3.13 ± 0.19^c^
α‐Pinene	1.96 ± 0.19^c^	3.92 ± 0.34^c^	1.96 ± 0.13^c^	1.96 ± 0.15^b^
Limonene	15.68 ± 3.21^d^	15.68 ± 2.97^d^	3.92 ± 0.21^d^	3.92 ± 0.14^c^
Citral	0.50 ± 0.07^a^	15.84 ± 3.82^d^	0.99 ± 0.11^b^	0.99 ± 0.13^a^
Nonanal	0.48 ± 0.06^a^	15.36 ± 2.74^d^	0.48 ± 0.08^a^	1.92 ± 0.19^b^
Decanal	0.20 ± 0.01^a^	7.76 ± 1.32^c^	1.94 ± 0.15^c^	1.94 ± 0.16^b^
Linalool	0.98 ± 0.08^b^	3.92 ± 0.31^c^	0.98 ± 0.11^b^	0.98 ± 0.13^a^
Terpineol	1.92 ± 0.23^c^	3.84 ± 0.27^c^	0.48 ± 0.07^a^	1.92 ± 0.12^b^

*Note*

Data are presented as mean ± standard deviation, *n *= 3. Different superscript letters represent the significant differences at *p* < 0.05.

Seven individual constituents were selected to test their antimicrobial activity (Table 2). Citral, nonanal, decanal, linalool, and terpineol had higher antagonistic activity (MIC = 0.20–1.98 μl/ml) against *S. cerevisiae*,* B. subtilis,* and *S. aureus* than Limonene and α‐pinene (MIC = 1.96–15.38 μl/ml). However, all tested individual constituents showed lower sensitivity against *E. coli* with MIC ranging from 3.92 to 15.84 μl/ml. Generally, the tested aldehydes and alcohols had better antimicrobial activities than monoterpene hydrocarbons. These results were consistent with the previous studies that some oxygenated compounds presented a higher antimicrobial activity than nonoxygenated hydrocarbons (Burt, 2004). Fisher et al. (Fisher & Phillips, 2006) demonstrated that linalool and citral in *Citrus* EOs had antimicrobial effects against *Campylobacter jejuni*,* E. coli* O157, *L. monocytogenes*,* B. cereus,* and *S. aureus*. Moreover, some researchers have shown that carvone and limonene oxide were active against a wide spectrum of pathogenic fungi and bacteria (Aggarwal et al., 2002; Mustafa, 2015). We noticed that Gannan navel orange light phase EO had higher content of carvone and limonene oxide than cold pressed EO which might due to oxidation during distillation. Higher activity of light phase EO might be attributable to its higher proportion of oxygenated compounds. Meanwhile, antimicrobial activity of both EOs might also be due to the synergistic, additive, and antagonistic interaction of their constituents (Burt, 2004). The investigation of the individual constituents in EO can provide useful information to fit different products or purposes with simultaneous elevation of their antimicrobial activities.

### 3.3 Effect of the environmental pH and temperature on antimicrobial activity of EOs

**Figure 1:**
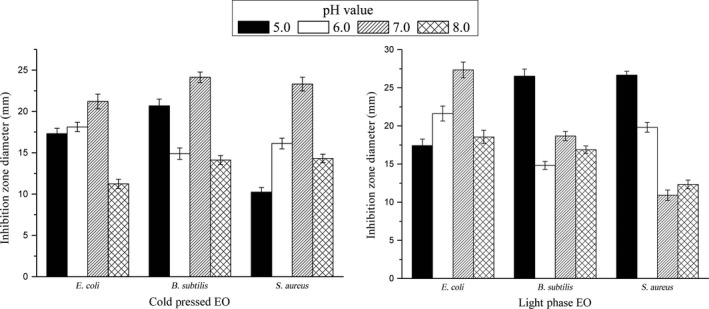
Antimicrobial activity of Gannan navel orange peel EOs at different pH values environment. Zone of growth inhibition values is presented as mean ± standard deviation (*p *< 0.05). The error bars represent the SEM of the replicates

The antimicrobial activity of cold pressed EO and light phase EO was closely related to pH value of the medium (Figure 1). The antibacterial activity was significantly different with increasing pH values. Cold pressed EO showed highest antibacterial activity against *E. coli*,* B. subtilis* and *S. aureus* at pH 7.0. Light phase EO showed strongest antibacterial activity against *B. subtilis* and *S. aureus* at pH 5.0, against *E. coli* at pH 7.0. Then, the inhibitory activity obviously decreased with the increase of alkalinity in the environment condition. These results suggested that Gannan navel orange EO showed best antimicrobial under pH value ranged from 5.0 to 7.0, which were consistent with previous studies that *Citrus* EOs showed high antimicrobial activity under the partial acidic condition (Bakkali et al., 2008; Burt, 2004). It was probable that under acidic condition, active constituents dissolved better in the lipid phase of the bacterial membrane, and their binding abilities with membrane protein enhanced, leading to a higher antimicrobial activity (Burt, 2004; Durairaj, Srinivasan, & Lakshmanaperumalsamy, 2009). In contrast, under alkaline condition, certain chemical reaction such as aldol condensation would occur among active oxygenated constituents especially aldehydes in EO, leading to a lower inhibitory activity.

**Figure 2:**
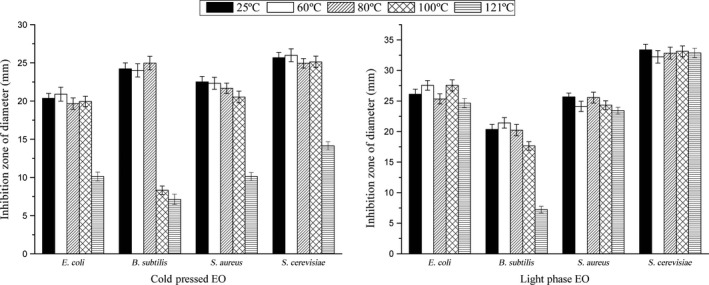
Antimicrobial activity of Gannan navel orange peel EOs at different heat treatment temperatures. Zone of growth inhibition values is presented as mean ± standard deviation (*p* < 0.05).The error bars represent the SEM of the replicates

The activity against *E. coli*,* S. aureus,* and *S. cerevisiae* showed no significant difference when EOs were heat‐treated at 60–100°C for 20 min, except that the activity against *B. subtilis* reduced nearly threefold after treatment at 100°C (Figure 2). When light phase EO was treated at 121°C for 20 min, its activity against *E. coli*,* S. aureus* and *S. cerevisiae* was not obviously different except that its activity against *B. subtilis* reduced nearly threefold. These results demonstrated Gannan navel orange EO have good thermal stability, especially for light phase EO at high temperatures. Thus, light phase EO was beneficial to use as antimicrobial agents for high‐temperature food processing.

With consumer trends for natural alternatives instead of synthetic chemical‐based antimicrobials and changes of legislation, *Citrus* EOs may provide a solution for both industry and consumers, as they are generally recognized as safe in food (Bakkali et al., 2008; Mustafa, 2015; Sharma et al., 2017). Exploitation of EOs as food preservative would be helpful to reduce waste and create new economic value. The findings of the present investigation that Gannan navel orange EO possessed a wide spectrum of antimicrobial activity and good thermal stability, especially for light phase EO, as some adverse compositions such as wax and pigment were removed by molecular distillation, may be beneficial to develop safe plant‐based preservatives in food industry.

## 4 CONCLUSIONS

Molecular distillation of cold pressed Gannan navel orange EO provided light phase EO, and the chemical composition and antimicrobial activity of both EOs were studied. Both EOs showed a wide spectrum of antimicrobial activity against Gram‐positive, Gram‐negative microorganisms, and yeast, with MIC values ranging from 0.39 to 3.13 μl/ml. Light phase EO had better antimicrobial activity than cold pressed EO. Oxygenated compounds might be important constituents to inhibit microorganisms. Light phase EO exhibited the best antibacterial activity under weak acidic and neutral conditions with a good thermal stability might be used as a novel antimicrobial agent in food industry.

## CONFLICTS OF INTEREST

The authors declare that they have no conflict of interest.

## ETHICAL STATEMENT

This article does not contain any studies with human participants or animals performed by any of the authors.
